# Family physician service quality and sustainability: a roadmap for Pakistan’s healthcare sector

**DOI:** 10.3389/fmed.2024.1455807

**Published:** 2024-12-05

**Authors:** Saifullah, Zhiqiang Ma, Mingxing Li, Muhammad Qasim Maqbool, Feng Chen

**Affiliations:** ^1^School of Management, Jiangsu University, Zhenjiang, China; ^2^Department of Management Sciences, University of Okara, Okara, Pakistan

**Keywords:** family medicine, quality and facility, professionalism, technology, community health, cooperation and care, sustainability

## Abstract

**Introduction:**

The number of family medicine consultants has increased during and after the COVID-19 pandemic. However, research on family medicine services specific to Pakistan remains limited. Therefore, this study aimed to explore family physician services in Pakistan.

**Methods:**

To meet the study goals, we collected data using snowball and purposive sampling. A questionnaire was used exclusively to collect data from family physician consultations. The data were examined using the SmartPLS structural equation model to test the study model’s reliability and validity.

**Results:**

The study findings showed that using resource utilization and allocation, utilization of technology, professionalism improvement, medical attention, cooperation, and caring were positively significant to employee welfare and assistance in family medicine services. These dimensions were also positively significant to community involvement and advocacy for the sustainable development of family medical services in Pakistan.

**Conclusion:**

The study concluded that effective resource utilization, professionalism, medical care, cooperation, and the evaluation of quality and outcomes are key factors in promoting the growth of family medicine services. These indicators may enhance staff satisfaction, community involvement, and family physician service sustainability.

## Introduction

According to the Alma-Ata Declaration, the family doctor system is essential to universal primary healthcare (1). Over 50 countries and regions have adopted this system with favorable results, attracting interest from governments and medical communities (2). Chinese studies have highlighted the importance and problems of creating a family doctor’s system. These studies also examined its implications for service consumption, management of non-communicable diseases, medical expenditures, and patient satisfaction (3–6). In Eastern countries, especially emerging ones, the family doctor model is experiencing a comeback (7).

The benefit of family doctors lies in their unique ability to work closely with families, ensuring they can truly look out for their patients’ best interests when it comes to health problems. They are also skilled at recognizing when to refer patients to specialists, community resources, and other healthcare services (8). The introduction of family medicine could make the healthcare system more effective. Other benefits of including family medicine in the healthcare system are that it saves money and is better for individuals and the country as a whole (9). The World Health Organization (WHO) estimates that increasing PHC interventions in low- and middle-income countries might save 60 million lives and enhance life expectancy by 3.7 years by 2030 (10). The WHO reports that family doctors, general practitioners, and family doctors have the responsibility of delivering comprehensive healthcare services and facilitating the provision of associated services by other healthcare professionals as needed (11).

In developed nations, such as the United Kingdom, the United States of America, Canada, Australia, and Japan, the family doctor system has undergone significant development (12, 13). Furthermore, China has concentrated on examining the importance and challenges associated with implementing a family doctor system. These studies have investigated the policy implications of family doctors concerning service utilization, management of non-communicable diseases, medical expenses, and patient satisfaction (3–6).

On the other hand, in the education system in Pakistan, medical schools offer limited training in family medicine, despite the fact that many of their graduates go on to become general practitioners (14). Family medicine provides primary care to individuals and families, across all age groups, from young children to the elderly. It focuses on the context of family and community, emphasizing the promotion of wellness and the prevention of illnesses (15). Family medicine should be taught to undergraduate medical students in Pakistan to foster interest in this increasingly critical specialty and improve medical services (16).

**Figure 1:**
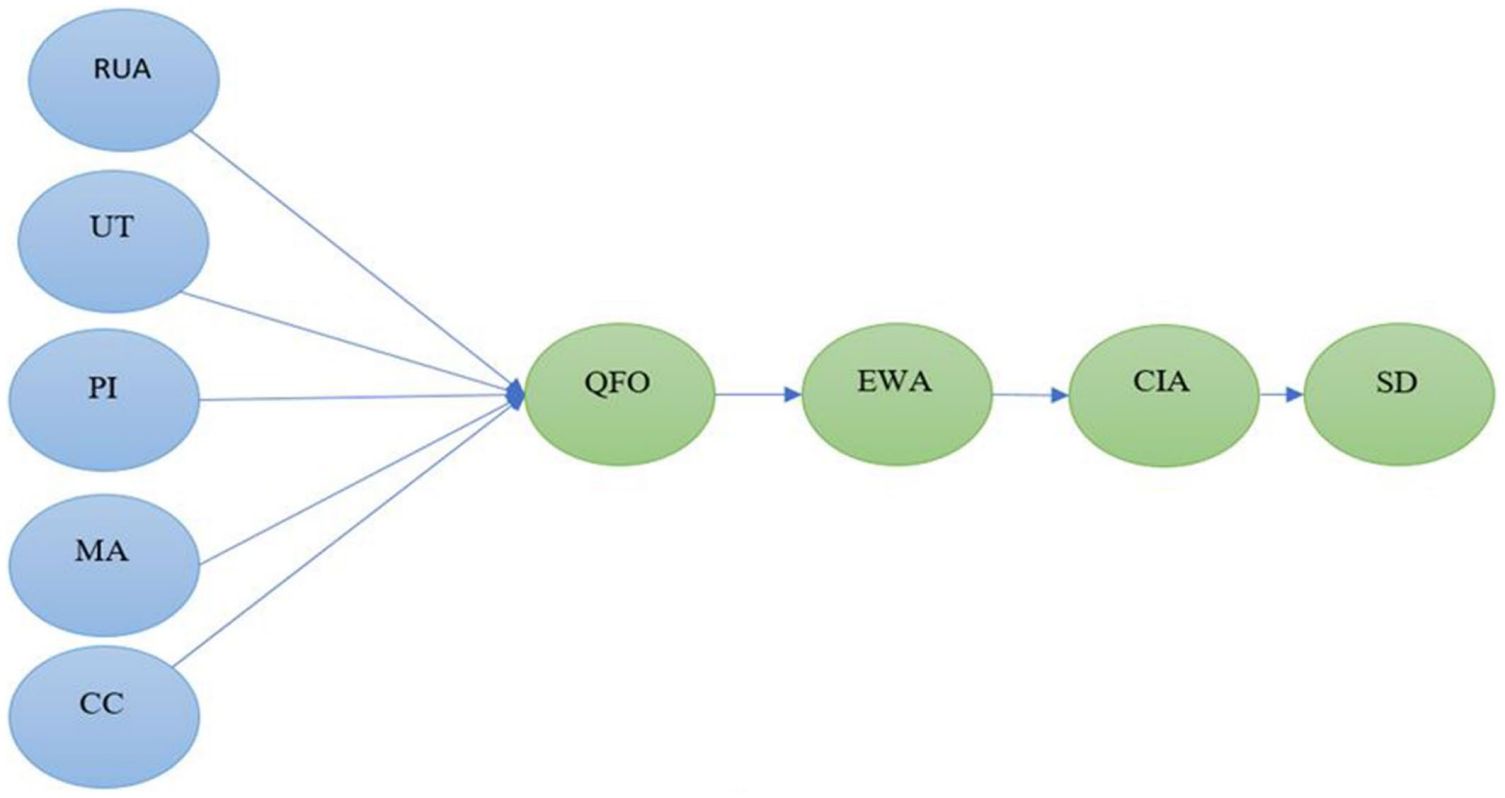
Conceptual model of the study. Figure 1 shows that RUA is for resource utilization and allocation; UT is for utilization of technology; PI is for professionalism improvement; MA is for medical attention; CC is for cooperation and caring; QFO is for quality and facilitating outcomes; EWA is for employee welfare and assistance; CIA is for community involvement and advocacy; and SD is for sustainable development.

However, research on family physicians lacking in developing nations leaves a gap for lower-income nations such as Pakistan, while family medical services can be crucial in providing reliable and convenient care in Pakistan. Hence, the objective of this study was to examine the quality of family physician services from the viewpoint of physicians and to develop a comprehensive model for service providers in Pakistan to improve their family medical service quality. The study’s model is illustrated in Figure 1.

## Theoretical background and hypothesis development

### Family medical services

The WHO recommends a high-performing, integrated primary healthcare (PHC) system to attain universal health coverage (UHC) and enhance health indicators (17, 18). Family doctors provide ongoing, comprehensive, empathetic, and personal care. They care for people of all ages and realize that health and sickness involve the mind, body, and spirit and depend on patients’ family and community lives (19–21). Medical expenses have increased, yet primary care physicians’ net income has fallen more than specialists’ net income (22). FM features are linked to patient satisfaction, health, and cost-service results. Accessibility, continuity, consultation duration, and doctor-patient relationship connection affected user satisfaction. Health improvements were linked to continuity, consultation time, doctor–patient connection, and preventive measures. Coordination of care has mixed health effects. In primary care, continuity, consultation time, doctor–patient communication, and prevention were cost-effective (23). If family medicine is introduced, it could make the healthcare system work better. Other benefits of including family medicine in the healthcare system are that it saves money and is better for individuals and the country as a whole (9). Family doctors dominate primary care in the UK, which is called the ‘jewel in the crown’ of the National Health Service (24). Moreover, family-centered care (FCC) fosters an honorable relationship among care providers and receivers (patients and their immediate social support network), advocates for a culture of patient safety and psychological wellbeing (1, 8), and additionally enhances healthcare and management competencies that lead to empowering the community (25, 26). According to the authors’ report, research on FCC has expanded beyond pediatric to adult acute, palliative, and end-of-life settings in the first 22 years of the 21st century. Patient safety, experience, and satisfaction are closely interconnected. Recent study includes patient involvement in FCC strategy formulation, health literacy interventions, and the adoption of telemedicine (27).

### Resource utilization and allocation

Health policymakers and health systems strive to achieve the critical objectives of equitable and efficient allocation of health resources and use of health services (28). Fairness in health service is a crucial objective pursued by health policymakers and health systems (29). The research on care resources in non-medical settings primarily examines the financial model of community-based care institutions (30) and the range of services offered to those with disabilities and the elderly (31). Furthermore, local case studies have analyzed the integration of family support and community care (32). Additionally, authors have explored the geographical availability of community care centers (33) and assessed the distribution of community-based senior learning resources, taking into account both demand and supply (34). Based on these factors, we attempted to evaluate the following hypothesis:

H1: There is a relationship between resource utilization and allocation and the quality and facility outcomes in family medicine.

### Utilization of technology

The importance of medical technology in healthcare quality was highlighted by a study (35). In addition, hygiene, sanitation, clinical skills, and the capacities of nurses are regarded as crucial aspects of providing treatment. The adoption of technology is significantly impacted by the quality of service (36). Medical technology is an important component of healthcare quality, but it is not the only factor. Hygiene, sanitation, clinical knowledge, and nursing abilities are also essential elements of standard care needs (35). Given the aforementioned considerations, we put up the subsequent hypothesis:

H2: There is a relationship between technology and quality and facility in the family medical service.

### Professionalism improvement

The significance of professionalism is of utmost relevance in both the domain of medicine and the procedure of medical education (37, 38). Jha et al. have categorized professionalism into many themes, such as adherence to values, patient accessibility, doctor–patient connection, demeanor, professional administration, self-awareness, and motivation (39). Three factors can influence the career advancement of general practitioners (GPs), with the most desirable path being a combination of a strong incentive mechanism, a strong professional identity, high success motivation, and high self-efficacy (40). Furthermore, the significance of emotional intelligence in physician–patient relationships is widely recognized and should be considered while demonstrating professionalism (30). Doctors are required to exhibit empathy through professional and polite communication that seeks to promote the patient’s involvement and compliance with medical recommendations (41–43). The revised literature incorporated the following hypotheses:

H3: There is a relationship between doctors’ professionalism and quality and facility outcomes in the family medical service.

H4: There is a relationship between doctor’s medical attention and quality and facility outcomes in the family medical service.

### Interpersonal cooperation and teamwork

Research has shown that working together and collaborating among healthcare providers can improve patient outcomes and improve access to healthcare (44–46). Research has shown that healthcare professionals who engage in collaboration with their peers are more efficient and experience higher levels of job fulfillment compared to those who do not (47–49). Multidisciplinary work refers to a process where different disciplines independently and simultaneously contribute to the same project, with a lower level of collaboration. It should not be mistaken for interpersonal interactions (50). Furthermore, it is imperative to grasp the concept of “collaboration” within the framework of interpersonal teams, as it has been emphasized as vital for assuring top-notch healthcare (50). Optimal interpersonal collaboration is attained by employing efficient communication and fostering a genuine appreciation for diverse perspectives within the team (51). A negotiated agreement between professionals that recognizes and appreciates the skills and contributions of different healthcare professionals in patient care is referred to as a “negotiated agreement between professionals which values the expertise and contributions that various healthcare professionals bring to patient care (p2) (52).” Therefore we developed the following hypothesis:

H5: There is a relationship between cooperation and caring and quality and facility outcomes in the family medical service.

### System quality and facilitating

The characteristics that contribute to system quality, as identified by DeLone and McLean (53), are system functionality, information system usefulness, timeliness, and interoperability. Research has shown that system quality has a considerable positive impact on consumer satisfaction with e-health services (54, 55). An analysis of the behavior of Pakistani Tele-Health users showed that the quality of the system significantly impacted their performance (56). Ineffective systems harm consumers’ willingness to adopt e-health services (55). However, subsequent investigations have contradicted the claims made by other researchers (57), as they found that system quality was deemed insignificant in the study. Thus, we put forward the subsequent hypothesis:

H6: There is an association between quality and facility improvement and employee welfare and assistance in family physician service.

### Employee welfare

Employers profit from offering paid leave, as it leads to enhanced employee productivity, decreased attrition, and reduced hiring and training expenses (58). Conversely, the absence of paid sick leave increases the likelihood of employees attending work while ill, which may facilitate the transmission of sickness to colleagues, resulting in heightened absenteeism and diminished productivity (59). According to the authors, welfare activities not only offer financial incentives but also enhance employees’ capabilities; improve their skills; facilitate the understanding of their challenges; provide allowances; enable the monitoring of working conditions; foster harmony through infrastructure; and establish frameworks for health and insurance against illnesses, accidents, and unemployment for employees’ families (60). This attempts to guarantee employee satisfaction and motivation inside the organization (61). The delaying of employee salary payments and associated welfare benefits may lead to apathy, thus undermining employee productivity, organizational creativity, and efficiency (62). Researchers investigated the impact of employee wellness facilities on performance outcomes (62). After revising this literature, we tried to test the following hypothesis:

H7: There is a relationship between employee well-being and community concern for family physical service sustainability.

### Community participation

Underprivileged individuals’ outcomes can be enhanced by community health workers (CHWs) (63, 64). Low-income and diverse patients are served by community health centers (CHCs) (65, 66). The goal of community health practice is to prevent disease in the community, detect and eliminate occupational and environmental hazards, and diagnose illnesses early (67). The quality of service is frequently influenced by the interactions occurring between customers and service providers (68, 69). Giving background support, the present study developed the dimensional model, as shown in Figure 1, and dimensional items are given in Table 1. The present study also proposed the following last hypothesis:

**Table 1:** Questionnaire dimension, items, and meaning.

Cooperation and caring	
CC1	Healthcare providers cooperate effectively and communicate proficiently in patient care.
CC2	Multidisciplinary care teams are utilized to address complex patient needs and enhance holistic treatment.
CC3	I encounter challenges in cooperating with others or participating in a multidisciplinary care team.
Community involvement and advocacy	
CIA1	Family physicians often participate in community participation and advocacy campaigns.
CIA2	Family physicians may advocate for policies and programs that benefit both patients and communities.
CIA3	Community engagement and campaigning for healthcare policy changes can be challenging.
Employee welfare and assistance	
EWA1	Initiatives are in place to assist family physicians’ wellbeing and resilience.
EWA2	Family physicians have appropriate professional and personal support to excel in their responsibilities.
EWA3	Experience severe stress or burnout due to workload or lack of assistance.
Medical attention	
MA1	Family physician services provide a high priority on patient-centered treatment, with a focus on accommodating patients’ needs and preferences.
MA2	Patients actively participate in the decision-making processes related to their medical care.
MA3	It can be difficult to involve patients in their care successfully or to remove barriers to patient engagement.
Professionalism improvement	
PI1	Family physicians have access to extensive educational and professional development opportunities.
PI2	I am encouraged to pursue further study and training to improve my expertise in family medicine services.
PI3	Access to relevant continuing education programs can be challenging.
Quality and facilitating outcomes	
QFO1	Quality improvement efforts aim to improve service delivery and patient outcomes.
QFO2	Effective family physician services can be measured and evaluated through established processes.
QFO3	Implementing quality improvement strategies and assessing outcomes in practice can be challenging.
Resource utilization and allocation	
RUA1	Is the allocation and utilization of resources, such as staff and equipment, in my practice efficient?
RUA2	Family physician services have sufficient funding and support for necessary resources.
RUA3	I face obstacles stemming from insufficient finance or lack of resources when providing high-quality healthcare services.
Sustainable development	
SD1	Family medicine tends to develop a competent workforce.
SD2	Family medicine improves the digital ecosystem and develops a national IT infrastructure to support family medicine.
SD3	Family medicine tends to develop training and education.
Utilization of technology	
UT1	To improve patient care, technology is skillfully used in family physical services.
UT2	In my profession, there are opportunities for innovation and the adoption of new technologies.
UT3	I experience obstacles in utilizing technology or do not have access to the required digital tools and platforms.

H8: There is an association between community association and support and the sustainable development of the family physician service.

## Methods

In this study, a cross-sectional descriptive design was used. The study targeted healthcare practitioners who provided family medical consultations in Pakistan’s different regions including Sindh, Punjab, and Khyber Pakhtunkhwa (KPK).

### Sampling and data collection

This study used non-probability sampling methods, chosen due to the lack of an appropriate sampling frame. To collect data, we used both snowball and purposive sampling methods. These methods were selected specifically to gather data from family physician consultants, as it was challenging to obtain a comprehensive list. First, we met with a family physician and requested them to fill out the research questionnaire and then we requested that you provide some family physician doctors’ contact information as we need to get a response from them for the study purpose. The family physician provided the family physician contact information. Then we contacted these physicians using different communication sources and requested them to fill out the research questionnaire. Thus in this way, the data were gathered to fulfill the research objectives. Previous research recommended mixed-method sampling as it mitigates bias and conserves time (70). The data were gathered from practitioners utilizing family physician services in Pakistan. The authors asserted that a minimum sample size of 200 observations is essential for establishing accurate fit measures in structural equation models (71, 72). This study collected 230 replies, of which 221 were deemed valid, while others were removed owing to inadequate information. Two master’s degree students were engaged to collect data independently to fulfill the study’s requirements. The lack of a comprehensive and formally recognized family medicine data list rendered the data collection process difficult and time-consuming, lasting approximately 5 months from October 2023 to February 2024.

### Survey questionnaire development

In this study, we developed research dimensions and items using the help of family medicine doctors. In the beginning, we searched the model of family medicine for sustainable development, which was lacking in the research contents. We finalized a few dimensions, and then we revised and amended these dimensions and items from the doctors. Ten doctors were proposed to revise the model’s dimensions and items. Eight of the ten doctors agreed with the model’s dimensions and items, while two suggested revising the items for sustainable development. After revision, two items of sustainable development were delivered again to update the model dimensions and items. All of the doctors agreed on the dimensions and items, the model was completed. In doing so, the questionnaires were created. The questions were divided into two sections: personal information (gender, age, education, and experience) and questions rated on a seven-point Likert scale. Table 1 shows the dimensions and items of the questionnaire. Ten family physician services providers helped construct this questionnaire by evaluating its dimensions, items, substance, phrasing, sequence, design, and difficulty. The questionnaire was edited twice by all participants for corrections. When all of the participants were satisfied and agreed with the aspects and items, the survey was carried out. The questionnaire’s dimensional items are given in Table 1.

#### Statistical method

After data collection, the statistical analysis was conducted using the SmartPLS program version 0.4 for data analysis and model testing. Researchers used SmartPLSM-4 bootstrapped data to find the significant levels of the path coefficient, loadings, and weights (73). According to a previous study (74), first, we checked whether the measurement model is accurate and trustworthy; then, we looked at the structural model’s connections.

## Results

### Personal information

This study investigated the family physician approach in Pakistan. The model was evaluated based on the participation of family physicians. There were 221 participants, with 36.1% identifying as female and 63.8% as male. The responses varied by age, with 7.2% of participants aged between 20 and 30 years old, 45.2% aged between 30 and 40 years old, 36.1% aged between 40 and 50 years old, and 11.3% aged over 50 years. All of them had qualifications, with 54.6% holding an MBBS, 37.5% holding a Doctor of Medicine (MD), and 8.1% holding a Bachelor of Ayurveda Medicine and Surgery (BAMS). Respondents had varying levels of experience as follows: 6.7% worked for 1–5 years, 16.0% for 6–10 years, 33.4% for 11–15 years, 36.1% for 16–20 years, and 6.7% had more than 20 years of experience (Table 2).

**Table 2:** Personal information (*N* = 221).

		Frequency	Percentage %
Gender	Female	80	36.1
	Male	141	63.8
Age (years)	20–30	16	7.2
	30–40	100	45.2
	40–50	80	36.1
	Above 50	25	11.3
Education	MBBS	120	54.6
	MD	83	37.5
	BAMA	18	8.1
Experienced (years)	1–5	15	6.7
	6–10	37	16.7
	11–15	74	33.4
	16–20	80	36.1
	Above 20	15	6.7

#### Construct reliability and validity

**Figure 2:**
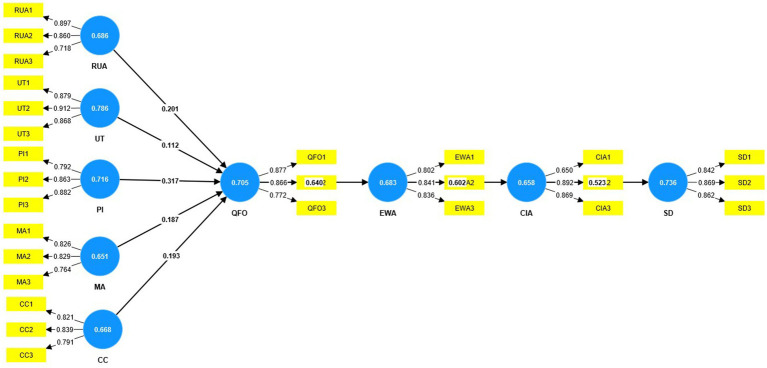
Structure model (algorithm).

Table 3 displays the appropriate values of CR, AVE, and outer loadings, which indicates convergent validity. According to the results of Hair et al. (75), convergent validity is demonstrated when the CR is greater than 0.50, the AVE is greater than 0.70, and the outer loadings are greater than 0.60. The model of the PLS algorithm is given in Figure 2.

**Table 3:** Construct reliability and validity.

Dimensions and Items	Loadings	Alpha	Reliability	AVE	*p*-value	VIF
Cooperation and caring		0.753	0.858	0.668		
CC1	0.821				0.000	1.544
CC2	0.839				0.000	1.499
CC3	0.792				0.000	1.491
Community involvement and advocacy		0.729	0.850	0.658		
CIA1	0.650				0.000	1.176
CIA2	0.892				0.000	2.283
CIA3	0.869				0.000	2.144
Employee welfare and assistance		0.768	0.866	0.683		
EWA1	0.802				0.000	1.431
EWA2	0.841				0.000	1.685
EWA3	0.836				0.000	1.674
Medical attention		0.736	0.848	0.651		
MA1	0.825				0.000	1.370
MA2	0.830				0.000	1.603
MA3	0.764				0.000	1.477
Professionalism improvement		0.801	0.883	0.716		
PI1	0.792				0.000	1.470
PI2	0.863				0.000	1.965
PI3	0.882				0.000	2.098
Quality and facilitating outcomes		0.789	0.877	0.705		
QFO1	0.878				0.000	2.115
QFO2	0.867				0.000	2.000
QFO3	0.769				0.000	1.387
Resource utilization and allocation		0.765	0.867	0.686		
RUA1	0.897				0.000	2.539
RUA2	0.860				0.000	2.366
RUA3	0.718				0.000	1.242
Sustainable development		0.821	0.893	0.736		
SD1	0.842				0.000	1.601
SD2	0.869				0.000	2.112
SD3	0.862				0.000	2.056
Utilization of technology		UT	0.864	0.917		
UT1	0.879				0.000	2.094
UT2	0.912				0.000	2.714
UT3	0.867				0.000	2.155

In addition, convergent validity can only be guaranteed if the AVE value is higher than 0.50 (74). The study’s AVEs are valid over 0.50, which means the results can be trusted (see Figure 2).

**Figure 3:**
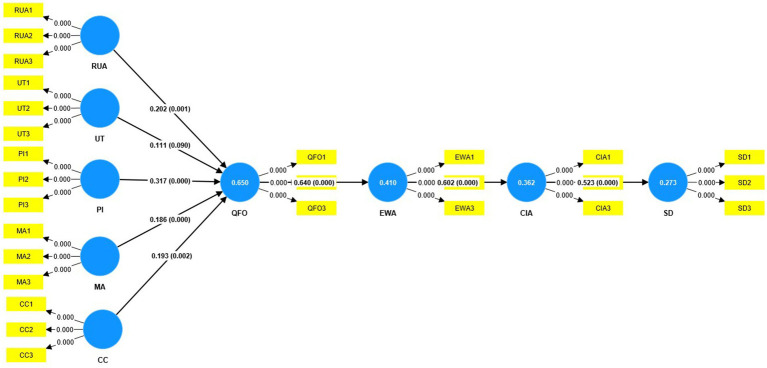
Structure model (bootstrapping).

### Discriminate validity

Criteria are typically used to quantify the shared variance of the latent variables in the model (76). The convergent validity of the measurement model can be evaluated using AVE and CR. Discriminate validity can be assessed using the Fornell–Larcker and cross-loading measures. Table 4 shows that off-diagonal values of each construct are found to be smaller than the square roots of AVE, hence satisfying the validity of the Fornell–Larcker condition.

**Table 4:** Fornell–Larcker criterion.

	CC	CIA	EWA	MA	PI	QFO	RUA	SD	UT
CC	0.817								
CIA	0.505	0.811							
EWA	0.709	0.602	0.827						
MA	0.447	0.482	0.576	0.807					
PI	0.494	0.609	0.614	0.599	0.846				
QFO	0.580	0.487	0.640	0.647	0.697	0.839			
RUA	0.447	0.426	0.493	0.587	0.513	0.626	0.828		
SD	0.419	0.523	0.424	0.527	0.520	0.547	0.626	0.858	
UT	0.510	0.458	0.568	0.593	0.623	0.637	0.591	0.578	0.886

Henseler and Sarstedt (77) developed the HTMT correlation, which serves as a robust measure of discriminate validity. To establish validity, the HTMT value must be below 0.90 (78). All of the buildings had HTMT ratios lower than 0.90, as shown in Table 5. This provides evidence that the model is worthwhile and that the constructs are sufficiently valid.

**Table 5:** Heterotrait–Monotrait ratio (HTMT) matrix.

	CC	CIA	EWA	MA	PI	QFO	RUA	SD	UT
CC									
CIA	0.672								
EWA	0.920	0.798							
MA	0.606	0.651	0.753						
PI	0.637	0.786	0.781	0.770					
QFO	0.748	0.640	0.823	0.834	0.877				
RUA	0.590	0.577	0.642	0.771	0.660	0.805			
SD	0.537	0.677	0.531	0.667	0.640	0.679	0.784		
UT	0.629	0.570	0.694	0.736	0.749	0.772	0.728	0.685	

RUA is for resource utilization and allocation; UT is for utilization of technology; PI is for professionalism improvement; MA is for medical attention; CC is for cooperation and caring; QFO is for quality and facilitating outcomes; EWA is for employee welfare and assistance; CIA is for community involvement and advocacy; SD is for sustainable development.

### Coefficient of determination (R2)

There are several factors to take into consideration, including the total impact size and variance created by independent variables, as well as the prediction accuracy of the model as measured by R2. The CIA explained 36.2% of the variation, as indicated by R^2^ of 0.36%, attributed to employee welfare and assistance. Despite EWA exhibiting a variation of 0.410, its R2 value of 0.41% is attributable to quality and facility outcomes. Similarly, the study demonstrates that a QFO R2 of 0.651 signifies that 0.65% is attributable to (RUA, UT, PI, MA, and CC), whereas an SD R2 of 0.273 implies that 0.27% is accounted for Table 6. It presents a valuable R2 linked as a satisfactory research framework.

**Table 6:** Coefficient of determination (R2).

	R-square	R-square adjusted
CIA	0.362	0.359
EWA	0.410	0.408
QFO	0.651	0.643
SD	0.273	0.270

### Testing model (bootstrapping)

To determine the importance of the family medical model, the bootstrapping technique was used to compute significant values (79). Table 7 shows the bootstrapping results. We evaluated how the five variables affected the quality and facilitating outcomes (QFO), employee welfare and assistance (EWA), community participation and advocacy (CIA), and sustainable development. Table 7 confirms the study model is supported and accepted. First, we examined H1: the impact of resource utilization and allocation (RUA) on quality and facilitating outcomes (QFO), which shows positive outcomes RUA - > QFO (*β* = 0.202, T-stat = 3.399, *p* > 0.001), and second, we examined H2: the utilization of technology to impact on quality and facilitating outcomes is not significant UT - > QFO (*β* = 0.111, T-stat = 1.695, *p* > 0.090). Third, we also examined H3: the impact of professional improvement (PI) on quality and facility outcomes (QFO). The results showed highly supported PI - > QFO (*β* = 0.317, T-stat = 4.236, *p* > 0.000). Furthermore, we tested H4: the impact of medical attention (MA) on quality and facilitating outcomes (QFO) MA - > QFO (*β* = 0.186, T-stat = 3.771, *p* > 0.000), which is also positively significant. We examined H5: the effect of cooperation and caring (CC) on quality and facilitating outcomes - > QFO (*β* = 0.193, T-stat = 3.080, *p* > 0.002), which is greatly supported.

**Table 7:** Testing model (bootstrapping).

Hypothesis description		Path coefficient	T statistics	*p*-values	Conclusion
H1.	RUA - > QFO	0.202	3.399	0.001	Supported
H2.	UT - > QFO	0.111	1.695	0.090	Unsupported
H3.	PI - > QFO	0.317	4.236	0.000	Supported
H4.	MA - > QFO	0.186	3.771	0.000	Supported
H5.	CC - > QFO	0.193	3.080	0.002	Supported
H6.	QFO - > EWA	0.640	14.077	0.000	Supported
H7.	EWA - > CIA	0.602	10.799	0.000	Supported
H8.	CIA - > SD	0.523	9.949	0.000	Supported

The significance level is *p* < 0.001 and *p* < 0.05.

Furthermore, we tested H6: the impact of quality and facility outcomes (QFO) on employee welfare and assistance (EWA). The results showed highly significant QFO - > EWA (β = 0.640, T-stat = 14.077, *p* > 0.000). In addition, we tested H7: employee welfare and assistance (EWA) and community involvement and advocacy (CIA) EWA - > CIA (*β* = 0.602, T-stat = 10.799, *p* > 0.000), which reveals a positive and substantial link between both. Similarly, we finally tested H8: community involvement and advocacy (CIA) and sustainable development (SD) CIA - > SD (β = 0.523, T-stat = 9.949, p > 0.000), which is highly significant.

## Discussion

According to the authors, family doctors work with families because they are the only ones who can properly watch out for their patients’ health needs and refer them to specialists, community resources, and health services (8). Other benefits of family medicine include cost savings and improved health outcomes which are advantageous both for individuals and for the country (9). Our initial H1 reveals that resource utilization and allocation (RUA) can significantly improve family medicine service quality and facility outcomes (QFO). Health policymakers and systems aim for equality and efficiency in resource allocation and service use (28). Every health service needs resources. Health policymakers and systems should therefore prioritize health service equity (29). Non-medical community care resource research should focus on the economic model of community-based care institutions (30) and services for disabled and elderly people (31).

Second, H2 concerning the influence of the utilization of technology (UT) on quality and facility outcomes for family medicine services in Pakistan demonstrates a poor significant level of validity. Other studies have validated the use of online services. According to several studies, telemedicine works in America, Southeast Asia, and Europe (80). Its effectiveness can be demonstrated in underdeveloped areas with limited doctors, health facilities, and distances (81). Healthcare quality depends on medical technology (35). Service quality greatly influences the adoption of technologies (36). As a developing nation with a limited healthcare budget, Pakistan may face a technology deficit, making this study less significant.

Third, we examined H3 concerning professionalism improvement (PI) and family medical service quality and facility outcomes (QFO). It has long been understood that professionalism is essential to both medical practice and medical education (37, 38). Jha et al. have identified compliance with values, patient access, doctor–patient relationship, demeanor, professional management, personal awareness, and motivation as the themes of professionalism (39). In physician–patient relationships, emotional intelligence is acknowledged as crucial (40) and must be taken into account when practicing professionalism. Doctors must show empathy by communicating professionally and courteously to encourage patient participation and medical advice (41–43).

Moreover, our hypotheses (H4 and H5) regarding medical attention (MA) and cooperation and caring (CC) affect family medicine service quality and facility outcomes (QFO), which is an important element in family physician content. According to a study on medication-related doctor–patient communication, patients were not typically involved in consultations or provided information about their prescriptions (82). The patient–provider contact may also be impacted by a wide range of other variables, such as cultural differences, gender, and attitudes (83). Enhancements in patient–physician communication and counseling have the potential to direct patients toward attainable preoperative expectations (84), and interpersonal communication and teamwork improve patient outcomes and healthcare access (44–46). Collaboration boosts productivity and job satisfaction in healthcare (47–49).

Furthermore, our hypothesis (H6) regarding the quality and facilitating outcomes (QFO) for employee welfare and assistance (EWA) is strongly supported in the study model. The study’s conclusions, which are in line with earlier research, imply that aspects of care, empathy, a positive working relationship, and attention to needs may be crucial elements of what it means to regard patients as unique individuals (85, 86). To address patient needs, doctors should know family privacy, listen to patients, involve them in decision-making, use evidence-based protocols, and prioritize patients. According to a previous study (53), system quality depends on functionality, information system usefulness, timeliness, and interoperability. SYQ considerably improves e-health consumers’ happiness, according to research (54, 55). In the family physician systems, offering telehealth services may improve the quality and convenience of the service system for providers and recipients. Recently, a study suggested that telehealth services reduce travel time and expenses, provide convenient treatment options, and lead to changes in healthcare payments, infrastructure, and staffing (87). Thus, using e-health services in family medicine could enhance quality and facility for receivers and providers.

This study also supports H7 concerning community engagement and advocacy (CI&A). Community involvement and advocacy are essential for family medical care sustainability. According to the authors, specific clinical characteristics of psoriatic arthritis (PsA) had a notable influence on the health-related quality of life, daily activities, and satisfaction with treatment for both patients and physicians. This emphasizes the significance of managing symptoms effectively to enhance the health-related quality of life for patients (88). Community health professionals improve underprivileged patients’ outcomes (63, 64).

In the final analysis, our research model proved the statistical relevance of community involvement and advocacy in family medical service sustainability (H8), which aids family medicine service research. Community health centers serve diverse, low-income individuals (65, 66). The WHO estimates that boosting PHC interventions in low- and middle-income countries could save 60 million lives and increase life expectancy by 3.7 years by 2030 (10). Family doctors, general practitioners, and other primary care providers play a crucial role in delivering comprehensive healthcare services and facilitating the provision of related services by other healthcare professionals (11). Furthermore, community health professionals have been shown to improve underprivileged patients’ outcomes (63, 64). Moreover, family doctors care for individuals of all ages, focusing on the overall health of patients their families, and the community (19–21).

## Conclusion

Family medical services are the foundation of healthcare, providing personalized and comprehensive care to individuals and families throughout life. Family physician services foster trust, understanding, and continuity of care through the patient-centered approach. This study found that resource utilization and allocation, technology use (UT), professionalism improvement (PI), medical attention (MA), and cooperation and caring (C&C) are most important for improving quality and facilitating outcomes (QFO) of Pakistani family medicine services. All of these characteristics improve employee welfare and assistance (EWA) for community engagement and advocacy (CI&A), which helps family medical services thrive. Family medical care depends on quality and professional resource use. The study found that improving the above factors can advance the family medicine approach. Family medical services prioritize companionship, professionalism, and community engagement, with a focus on improving the health of individuals, families, and communities. Finally, this study suggests that future research should incorporate additional dimensions of financial resources and patient-related aspects in family medical care.

## Limitations

This study has several limitations. First, this study was conducted in Pakistan, which can be used in other countries by using or adding different dimensions. Second, the study produced a model that allows for the development and addition of variables and dimensions. Furthermore, this study employed a structural equation model, but alternative methodologies could be used to enhance the robustness of the findings. Future research could focus on family physician service-based strategies, utilizing the capabilities of researchers in this field. Finally, this type of research requires funding, and the researcher may face several challenges while gathering information related to the study topic, especially in developing nations.

## Data Availability

The datasets presented in this article are not readily available because data for the study can not be shared. Requests to access the datasets should be directed to Saifullah; hakrosaifullah2@gmail.com.
